# The structural basis for high affinity binding of α1-acid glycoprotein to the potent antitumor compound UCN-01

**DOI:** 10.1016/j.jbc.2021.101392

**Published:** 2021-11-07

**Authors:** Erik J.B. Landin, Christopher Williams, Sara A. Ryan, Alice Bochel, Nahida Akter, Christina Redfield, Richard B. Sessions, Neesha Dedi, Richard J. Taylor, Matthew P. Crump

**Affiliations:** 1 School of Chemistry, University of Bristol, Bristol, UK; 2 BrisSynBio, University of Bristol, Bristol, UK; 3 Department of Biochemistry, University of Oxford, Oxford, UK; 4 School of Biochemistry, University of Bristol, Bristol, UK; 5 Discovery Sciences, UCB Biopharma, Slough, UK

**Keywords:** AGP2, UCN-01, staurosporine, X-ray crystallography, glycoprotein, kinase inhibitors, pharmokinetics, AGP, alpha-1-acid glycoprotein, AGP2-FL, full-length AGP2, AMT, amitriptyline, CDK2, cyclin dependent kinases, Chk1, Checkpoint 1, CPZ, chlorpromazine, DSP, disopyramide, TROSY-HSQC, transverse relaxation optimized - heteronuclear single quantum coherence spectroscopy

## Abstract

The α1-acid glycoprotein (AGP) is an abundant blood plasma protein with important immunomodulatory functions coupled to endogenous and exogenous ligand-binding properties. Its affinity for many drug-like structures, however, means AGP can have a significant effect on the pharmokinetics and pharmacodynamics of numerous small molecule therapeutics. Staurosporine, and its hydroxylated forms UCN-01 and UCN-02, are kinase inhibitors that have been investigated at length as antitumour compounds. Despite their potency, these compounds display poor pharmokinetics due to binding to both AGP variants, AGP1 and AGP2. The recent renewed interest in UCN-01 as a cytostatic protective agent prompted us to solve the structure of the AGP2–UCN-01 complex by X-ray crystallography, revealing for the first time the precise binding mode of UCN-01. The solution NMR suggests AGP2 undergoes a significant conformational change upon ligand binding, but also that it uses a common set of sidechains with which it captures key groups of UCN-01 and other small molecule ligands. We anticipate that this structure and the supporting NMR data will facilitate rational redesign of small molecules that could evade AGP and therefore improve tissue distribution.

The alpha-1-acid glycoprotein (AGP) is an acute phase drug-binding transport protein that is abundant in the blood plasma (1). AGP is produced predominantly hepatically but is significantly upregulated in proinflammatory conditions where its expression can be driven by other cells and tissues including immune and endothelial cells and adipose tissue (2). AGP expression is itself controlled by major regulatory mediators that include IL-6, IL-1, and TNF-α, and the blood plasma levels can rise five-fold (∼5 mg/ml) compared with circulating levels in healthy individuals (3). The raised AGP2 levels have been associated with a range of pathological conditions including cancer, infection, neuroinflammation, and cardiovascular diseases (4, 5, 6, 7), but AGP’s true physiological role remains unclear. AGP’s activities are associated with immunomodulation and small molecule transport, but the link between the binding of natural ligands (*e.g.*, serotonin, histamine, and melatonin) and AGP function is not understood (8). To date, the number of endogenous and exogenous ligands (*e.g.*, drugs) of AGP run into the hundreds and is the subject of continued review (1, 7, 9, 10, 11).

Human plasma lipocalins present as two variants known as AGP1 and AGP2 (or F1/F2/S and A forms, respectively) (Fig. 1) (12). In their mature forms, both proteins are comprised of 183 aa residues with a total of 21 amino acid differences between them. AGP1/2 are heterogeneously glycosylated *in vivo* across five N-linked sites (Asn15, Asn38, Asn54, Asn75, and Asn85). Functionally, glycosylation reduces AGP clearance in the kidneys, increases solubility, and may sometimes modulate drug binding (13, 14, 15). AGP1 and AGP2 each exhibit a range of affinities (*K*_*D*_ ∼ 10^−5^–10^−9^) for endogenous and exogenous ligands which may be specific to one AGP variant or bind both with comparable avidity (16). For example, the analgesic methadone is selective for AGP2, whereas the oncology drug imatinib binds more strongly to AGP1 (17) and the antipsychotic thioridazine has comparable affinity for the two variants (16).

**Figure 1:**
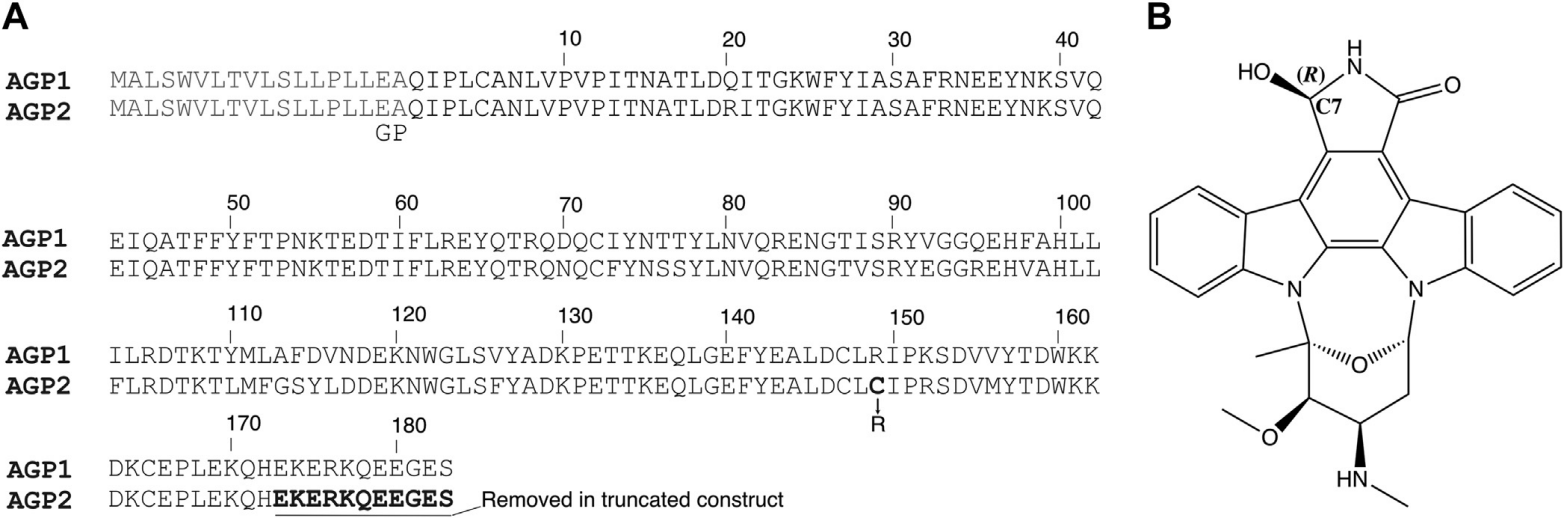
**Amino acid sequences of AGP1 and AGP2and structure of UCN-01.***A*, Sequence alignment of AGP1 and AGP2. The N-terminal 18 residues are cleaved in the mature protein, and the AGP2 construct in this study has an N-terminal GP extension after posttranslational cleavage of the His_6_-tag. The C-terminal truncation is highlighted in *bold*, and the C149R mutation previously reported was also included. *B*, the chemical structure of UCN-01. UCN-02 has the opposite (*S*) configuration. AGP, alpha-1-acid glycoprotein.

To determine their different ligand-binding specificities, high resolution crystal structures of nonglycosylated AGP1 (18) and AGP2 (14) ligand complexes have been solved revealing a lipocalin type fold-based around an archetypal eight-stranded β-barrel structure. The structure of AGP2 was solved in complex with three further ligands, disopyramide (DSP), amitriptyline (AMT), and chlorpromazine (CPZ) as well as a ligand-free state although this contained bound PEG (14). The AGP2 structures revealed that AGP2 has a smaller ligand-binding pocket and specific amino acid substitutions that might explain AGP2’s greater ligand selectivity than AGP1.

The structural studies of the AGP variants have not, however, yielded high-resolution structures of several medicinally important ligands. *In silico* docking studies have proposed a binding mode for the tyrosine kinase inhibitor and widely used leukemia treatment imatinib to AGP1 (19) but there is no high-resolution structure. There are also no data for staurosporine or its hydroxylated derivatives UCN-01 and UCN-02 (Fig. 1), which have been extensively investigated as antitumour compounds because of their ability to inhibit protein kinases involved in cell proliferation (20, 21). Staurosporine-bound protein kinase C and many other kinases showed high toxicity, precluding its direct use as a therapeutic. UCN-01 on the other hand was more selective and a potent inhibitor of protein kinase C (22), as well as the cell cycle kinase checkpoint 1 (chk1) and cyclin-dependent kinases (*e.g.*, CDK2). These properties led to its development as a therapeutic, but multiple clinical trials (*e.g.*, NCT00082017, NCT00036777, and NCT00004263) (23) were terminated because of lack of efficacy. It has subsequently been shown that UCN-01’s strong affinity for AGP2 has a significant negative impact on its efficacy, and this interaction is known to give UCN-01 a poor distribution volume (24). Nonetheless, UCN-01 has been long recognized as potentiating the effect of a number of cytotoxic agents and has also gained renewed interest as a cytostatic protective agent alongside the widely used chemotherapeutic 5-fluoro-uracil in breast cancer (25). These emergent properties have raised the importance of understanding UCN-01–AGP2 interactions.

Here, we present a crystal structure and solution NMR studies of a UCN-01–AGP2 complex. These new data reveal that UCN-01 binds with its aromatic system in a perpendicular orientation to those of AMT and DSP, but with some similarities to CPZ. However, the size and orientation of UCN-01 permits a more extensive array of favorable interactions to be formed within the AGP2-binding pocket and is accompanied by significant stabilization of the structure upon ligand binding. This structure alongside backbone chemical shift assignments of AGP2 will facilitate the studies of further ligands or UCN-01 analogues aimed at abrogating AGP-2 binding.

## Results and discussion

### Optimization of AGP2 for NMR and structural studies

AGP2 has not previously been structurally studied by NMR. In an initial attempt to obtain solution NMR spectra of sufficient quality for chemical shift assignment, we expressed nonlabeled and uniformly ^15^N-labeled full-length AGP2 (AGP2-FL) from *E. coli*. This construct incorporated a cleavable N-terminal His_6_-tag and the C149R mutation shown to improve solution homogeneity of deglycosylated forms used in crystallographic studies (14). We used a rapid dilution-refolding strategy to form the two disulphide bridges (Cys 5 - Cys 147 and Cys 72 – Cys 165) as opposed to cytoplasmic folding methods (26). After refolding and removal of the N-terminal His_6_-tag, the analytical size-exclusion chromatography indicated the presence of a ∼22 kDa species (Fig. S1) consistent with monomeric, nonglycosylated protein with no indication of aggregation. A ^1^H-^15^N transverse relaxation optimized spectroscopy - heteronuclear single quantum coherence (TROSY-HSQC) spectrum of ^15^N-labeled material was collected at 298 K but was of poor quality as judged by the presence of less than 50% of the expected ^1^H-^15^N correlations and inhomogeneous peak intensities (Fig. S1). This suggested that the poor spectral quality might arise from misfolding or conformational averaging in the nonligated state. The protein production in *E. coli* results in loss of glycosylation and although the deglycosylated form has been the only robust preparation for successful structural studies (14) it was not clear at this stage if their absence was also influencing the solution behavior of AGP2 in the nonligated state.

Addition of a 2-fold excess of UCN-01 to AGP2-FL, however, yielded a much improved ^1^H-^15^N TROSY-HSQC spectrum, with a higher proportion of the expected number of resonances and more uniform peak homogeneity. AGP2-FL was therefore most likely correctly folded, and UCN-01 was able to bind and stabilize the structure as has been observed in other lipocalins (27). The NMR spectrum, however, remained of less than satisfactory quality (Fig. S1).

In previously reported ligand-bound AGP2 structures, a short C-terminal helix situated away from the ligand-binding pocket was observed with varied degrees of structure (14). The removal of partially structured residues might improve the solution behavior of AGP2 so a second construct, hereon referred to as simply AGP2, lacking 11 C-terminal residues (Fig. 1) was designed. AGP2 was expressed, purified, and refolded (Fig. S2) using the same method as the full-length protein and the N-terminal His_6_-tag cleaved. Analytical gel filtration confirmed monomeric protein, and formation of the two disulphide bridges was confirmed by treatment of AGP2 with iodoacetamide under reducing or oxidizing conditions and analysis by electrospray mass spectrometry (Fig. S2).

The addition of UCN-01 to AGP2 resulted in a significant increase in the melting temperature shifting by 13.7 °C from 61.9 °C to 75.6 °C, again indicative of high affinity binding of UCN-01 and thermal stabilization of the truncated form (28, 29) (Fig. S2). A ^15^N-labeled AGP2 sample in complex with UCN-01 also gave high-quality ^1^H-^15^N HSQC NMR spectra with close to the expected number of resonances (Figs. S3 and 2). In the absence of ligand, the spectral quality of the free AGP2 was again quite poor and together, these data suggest significant stabilization of the AGP2 core upon UCN-01 binding.

**Figure 2:**
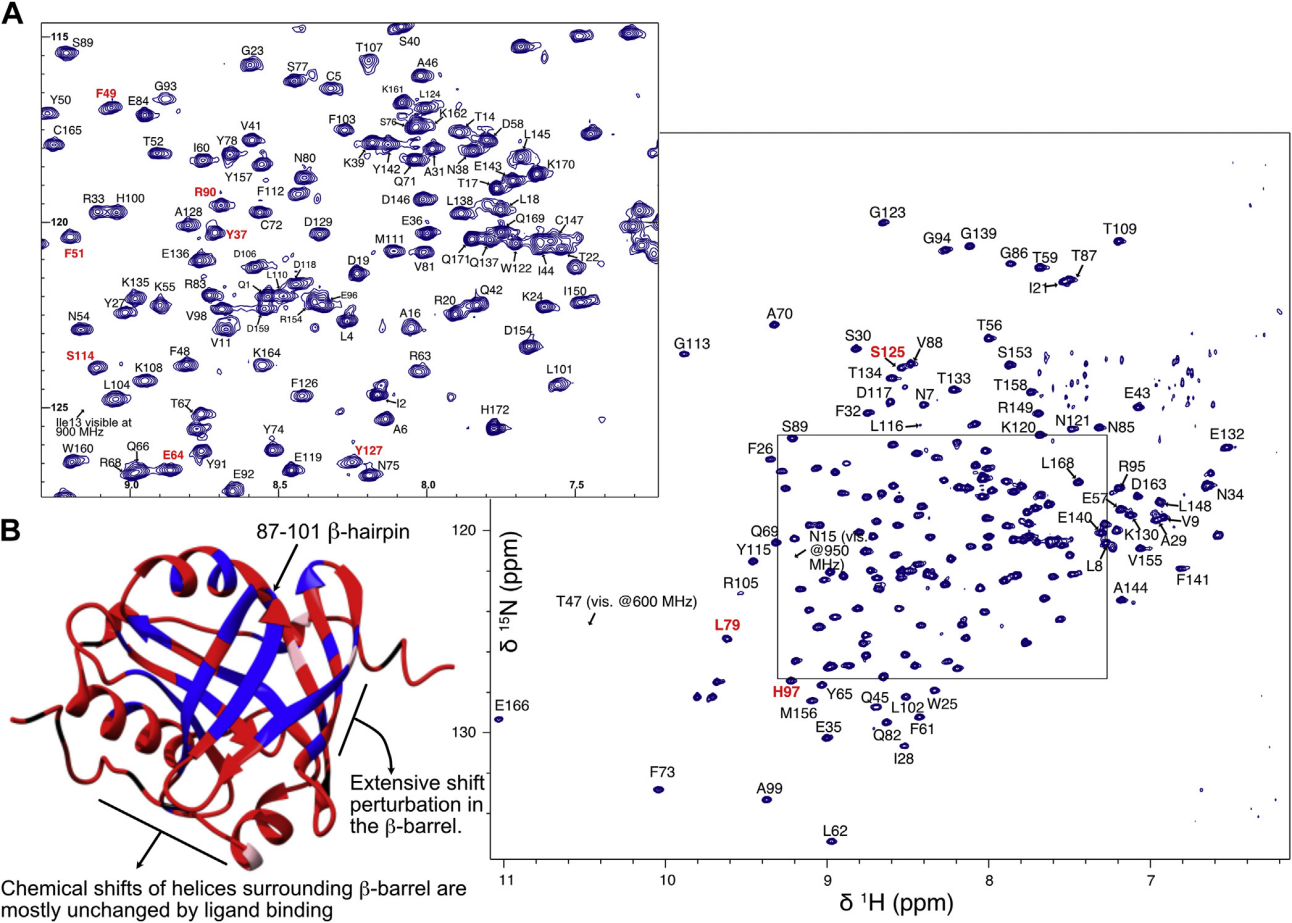
^**1**^**H-**^**15**^**N TROSY-HSQC spectrum of AGP2 bound to UCN-01 at pH 6.5 collected at 298 K at 700 MHz with complete backbone assignments.***A*, complete spectrum with peripheral assignments and expanded central region of the spectrum with assignments. The residues in the binding region of UCN-01 are highlighted in *red*. *B*, the structure of AGP2 with colored residues based on whether the backbone N-H peaks are observed (*red*) or not (*blue*) in the free AGP2 spectrum based on the UCN-01 spectrum. Proline residues are highlighted in *black* and residues only visible at different field strengths are highlighted in *pink*. AGP, alpha-1-acid glycoprotein; TROSY-HSQC, transverse relaxation optimized - heteronuclear single quantum coherence spectroscopy.

#### Global comparison of free and bound forms of AGP2

The NMR properties of the free AGP2 are consistent with the lack of crystallographic data for a strictly free form of AGP. AGP has crystallized in the free form, although the buffer components, such as polyethylene glycol, occupy the binding pocket suggesting this binding may induce some form of rigidification that is critical for crystallization (14). To further compare changes between free and bound forms, a ^13^C/^15^N sample of AGP2 in complex with UCN-01 was prepared which yielded 98% complete backbone assignments using standard triple resonance experiments acquired at 700 and 950 MHz (Fig. 2). The free and 2:1 UCN-01:AGP2 HSQC data were then compared in a reverse minimal chemical shift approach, necessitated by the lack of backbone assignments for the very low quality free AGP2 spectra. Residues that shifted most significantly included those directly involved in ligand binding, for example, Phe112 (14) (Figs. 2*B* and S3). However, residues distal to the binding site (such as Leu101) were also strongly affected and H-N pairs that form the H-bonding network of adjacent antiparallel β-strands. Therefore, a picture emerges of significant conformational rearrangement of free-AGP2 centered around deformation of the β-barrel architecture. There was a far less observable effect on the surrounding structural helices. In the absence of characterization of AGP2 modified with homogeneous glycosylation patterns, however, the precise nature of conformational sampling of this form is likely to remain unclear (15).

NMR dynamics studies can provide an indicator of the structuring, and internal motions of a macromolecule and main chain amide ^15^N relaxation rates for proteins and peptides are widely applied to detect them (30). Broadening of resonance peaks has been observed for the apo form of the fluorescein binding lipocalin FluA, and internal dynamics determined by NMR have been proposed to be a key feature for ligand recognition (27). When bound to fluorescein, the FluA protein backbone showed significant rigidification. *T*_1_, *T*_2_, and NOE relaxation data were therefore collected on a UCN-01:AGP2 complex made in a 2:1 ratio. The analysis of these key parameters across the length of the protein revealed that the residues exhibit a relatively uniform set of values across the length of the protein. (Fig. S4). Both the N- and C- termini showed reduced T_1_/T_2_ ratios and reduced NOE typical of fast (sub nanosecond) internal motions, but there was no evidence of patches of reduced T_2_ values commensurate with μs-ms exchange motions. The relaxation parameters further suggest a uniformly rigid protein in the UCN-01 bound form.

### Crystal structure of AGP2 bound to UCN-01

**Figure 3:**
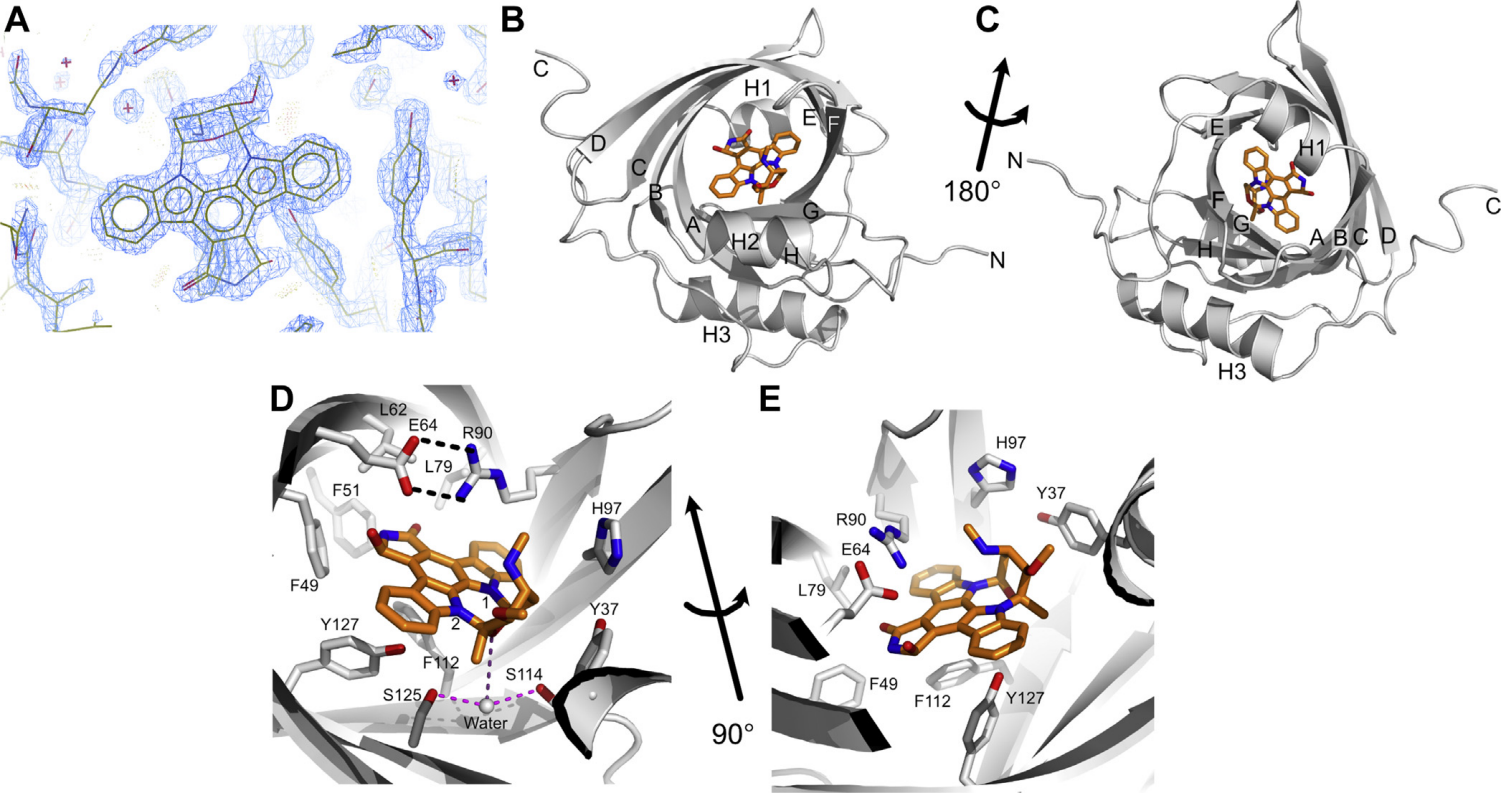
**Crystal structure of AGP2 in complex with the anti-tumor compound UCN-01.***A*, electron density for UCN-01 plotted at the RMSD (σ) level of 3.01 (0.8176 e/A^3^). *B*, cartoon representation of AGP2 in *gray* with UCN-01 (*orange*) bound in the central β-barrel. The β-sheets are labeled A–H, as annotated previously (14). The secondary structure was determined with STRIDE (45). *C*, as (*B*) but rotated ∼180°. *D*, close-up of UCN-01 bound in the β-barrel and (*E*) rotated ∼90°. The pocket consists of aromatic sidechains such as Phe49, a hydrogen-bonding interaction from Glu64 and potential cation-π interaction from Arg90. The N1 and N2 nitrogens of UCN-01 indole rings are labeled 1 and 2. AGP, alpha-1-acid glycoprotein. The bound water molecule is shown as a gray sphere.

Cocrystallization of UCN-01 with AGP2 yielded crystals diffracting to 1.82 Å representing the highest resolution achieved for an AGP2 complex to date. The structure was subsequently solved by molecular replacement using the structure of AGP2 bound to PEG [PDB: 3APU] and contained a single protein–ligand complex in the asymmetric unit. The electron density for UCN-01 in the AGP2-binding pocket was clearly resolved (Figs. 3*A* and S5).

The AGP2 structure has an archetypal eight-stranded antiparallel β-barrel structure that is characteristic of AGP and lipocalin folds (Fig. 3) (12, 18). This β-barrel, with strands labeled A-H, forms an internal cavity that defines the main ligand-binding pocket. Three helices, labeled H1-H3, are also present in the structure. Helix H1 (Asn15–Ile21) is N-terminal to the β-barrel, helix H2 (Glu35 – Gln42) intersects β-strands A and B, and helix H3 (Lys135 – Leu148) provides a C-terminal cap after strand H. The residues Gln166-Glu171 adopt a turn like structure rather than the short but regular fourth helix observed in the full-length construct (14).

UCN-01 makes multiple contacts with the binding pocket and sits in a plane almost perpendicular to the axis of the β-barrel (Fig. 3, *B*–*D*). Several aromatic residues line the binding pocket including Tyr37, Phe49, Phe51, His97, Phe112, and Tyr127 and form interactions with UCN-01 which is principally comprised of an extended aromatic ring system. Phe112 forms a CH-π interaction with the central benzene ring of UCN-01 and the Tyr127 hydroxyl group H-bonds to indole nitrogen N2. Neither Phe49 nor Phe51 form aromatic face to face or edge interactions with UCN-01. Together with Leu62 and Leu79, they instead sit, somewhat surprisingly, opposite the polar residues of the five-membered lactam ring. Glu64 and Arg90 sit above the plane of UCN-01 and form a salt-bridge connecting β-strands C and E. This carboxylate – guanidinium group interaction lies perpendicular to the UCN-01 aromatic system allowing the formation of a hydrogen bond from the lactam ring 7*R*-OH to Glu64 and a cation-π interaction with Arg90. The interaction between Arg90 and the UCN-01 aromatic system is of interest as π-stacking interactions are observed more frequently in published structures than T-shaped cation-π interactions, such as the one observed here (31). It is likely that the salt bridge with Glu64 residue influences the orientation of Arg90, making it more likely to adopt the observed conformation.

Tyr37 appears to form a parallel VdW interaction with one face of the oxy-bridged seven membered ring of UCN-01. A bridging water molecule is H-bonded equidistantly between Ser125 and Ser114 but beyond H-bonding distance (3.8 Å) from the bridging oxygen of UCN-01. This oxygen lacks any obvious H-bonding partner. Finally, the pendant methoxy and secondary amine groups both lie on the more solvent exposed face of UCN-01 and lack any discernible packing interactions.

### Comparison with other ligated AGP2 structures.

**Figure 4:**
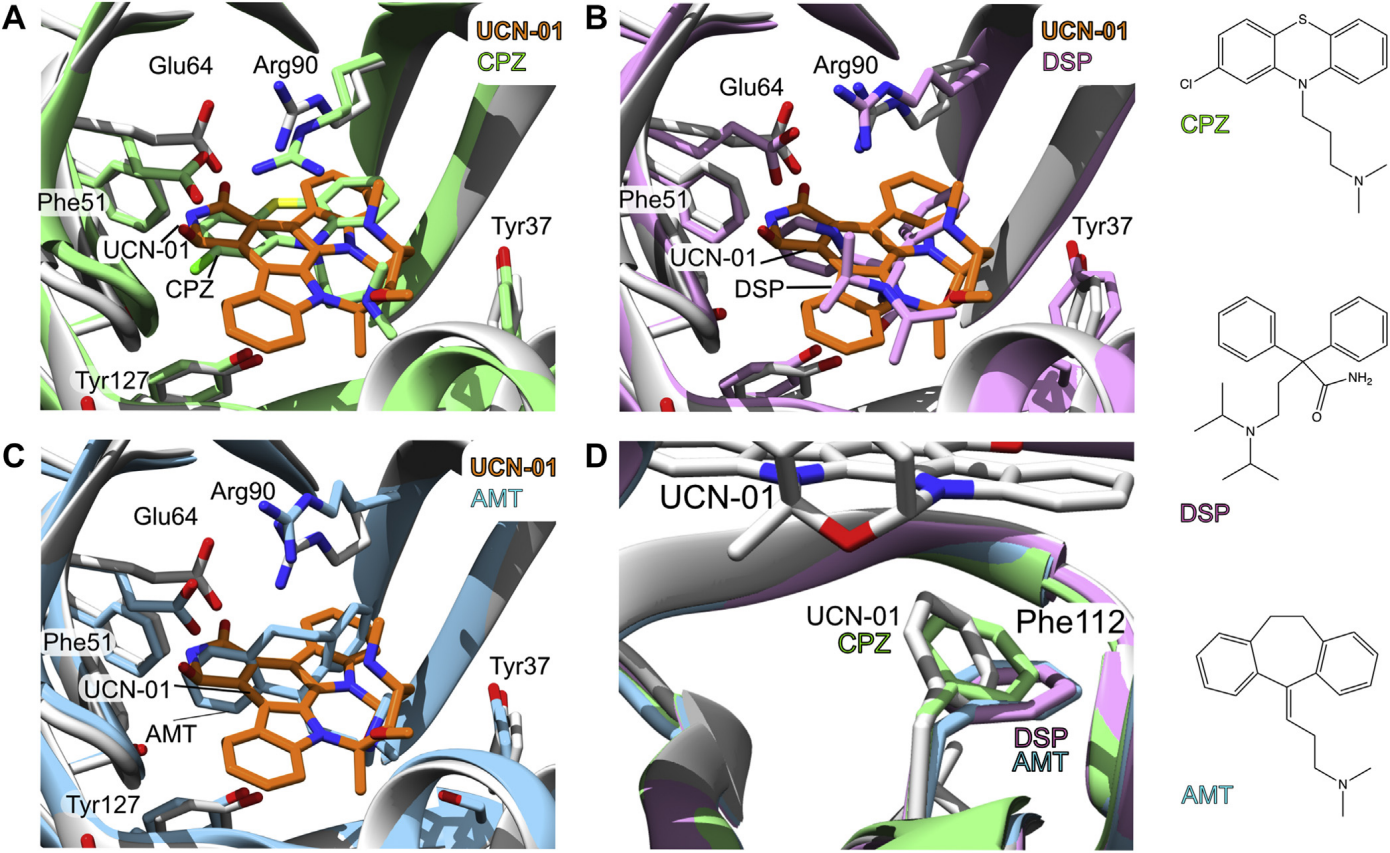
**Structural comparisons of all known AGP2–ligand complexes** (14)**.** All four ligands contain basic amine groups oriented toward the opening of the ligand-binding pocket. *A*, comparison of UCN-01 (*orange*) and CPZ (*green*) (PDB code: 3APX) binding to AGP2. The aromatic systems of the two ligands are observed in the same plane within the pocket and can form interactions with Arg90 on either side of the ring systems (*B*) comparison of UCN-01 and DSP (*pink*) (PDB code: 3APW) binding to AGP2. The ring system of DSP sits in a perpendicular plane to that of UCN-01 (*C*) comparison of UCN-01 and AMT (*blue*) (PDB code: 3APV). *D*, overlay of Phe112 conformation in AGP2 bound to UCN-01 with those of published complexes with CPZ, DSP, and AMT. AGP, alpha-1-acid glycoprotein; AMT, amitriptyline; CPZ, chlorpromazine; DSP, disopyramide.

Although solved at pH7.4, the UCN-01 bound AGP2 three-dimensional fold is very similar to previous ligated AGP2 structures solved at pH 4.6 (Fig. 4). Superimposing the backbone residues (11–167) of AGP2 bound to UCN-01 to DSP, AMT, and CPZ ligated structures gave RMSDs of 0.70, 0.62, and 0.81 Å, respectively. UCN-01 shares the same region of the binding pocket to these ligands and is buried within the β-barrel of AGP2. The opening to the binding pocket contains one of the N-linked glycosylation sites, Asn75, that would be present in native AGP2 and may also shape and define the ligand-binding propensity. This does not appear to be important for maintaining strong interactions with UCN-01 as observed with AMT, CPZ, and DSP (14).

The conformation of UCN-01 in the binding pocket is most like the drug CPZ. Both CPZ and UCN-01 are bound with aromatic ring systems in a similar plane (Fig. 4*A*) perpendicular to that of DSP and AMT (Fig. 4, *B* and *C*). CPZ, which otherwise binds in a similar fashion to UCN-01, interacts with Arg90 by a more typical stacking interaction and concomitantly Glu64 also changes in orientation. The π-systems of the two other ligands, for which crystal structures have been solved, are oriented perpendicular to those of UCN-01 and CPZ and so further comparisons of Arg90-π interactions are unavailable. The higher affinity (3.5 nM) of UCN-01 for AGP relative to CPZ may be explained in part by the more extensive aromatic ring system of UCN-01 which occupies parts of the binding pocket closer to Ile44. In the case of DSP, this pocket is occupied by aliphatic chains from the tertiary amine and with AMT, the pocket is occupied by acetate buffer.

Both UCN-01 and CPZ bind to AGP1 and AGP2 with comparable affinity (28) whereas AMT and DSP are specific for AGP2. The differences in ligand specificity for the two AGP variants is believed to be highly dependent on the differences at positions 112 and 114 (Leu112/Phe112 and Phe114/Ser114 in AGP1/AGP2 (14). Comparison with all four complexes shows that Phe112 is rotated toward the ligand plane of aromaticity in the case of CPZ and UCN-01, whereas it lies flat and is more restricted in the AMT and DSP complexes (Fig. 4*D*). Substituting Phe112 for Leu112 at this position may be accommodated by ligands binding in the mode of UCN-01 and CPZ but would clash with the orthogonal aromatic systems of AMT and DSP. Likewise, UCN-01 is also unlikely to clash with Phe114 if its AGP1-bound form is homologous to the AGP2–UCN-01 complex.

**Figure 5:**
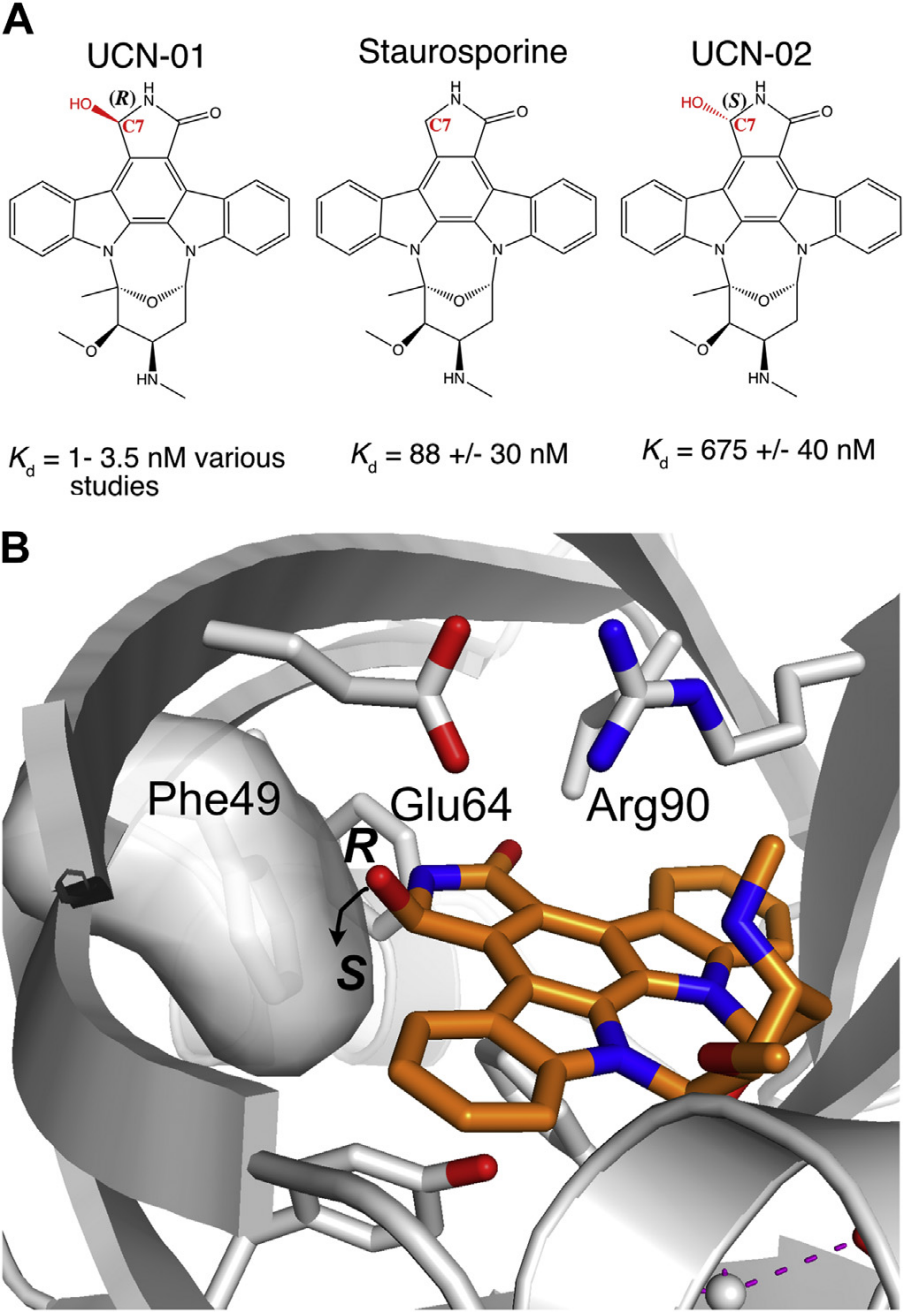
**Proposed structural basis for affinity of UCN-01, -02 and staurosporine for AGP2.***A*, chemical structures of staurosporine and UCN-01 and -02 derivatives showing switch in stereochemistry of the C7 hydroxyl group. *B*, potential clash with Phe49 (shown in space-filling) when C7 hydroxyl group is in the *S*-configuration (UCN-02).

UCN-01 is the highest affinity ligand for AGP2 determined to date with a *K*_d_ of 1 - 3.5 nM (28, 32). Interestingly, binding to UCN-01 is completely lost in rat AGP. Rat AGP1 has 44% identity to human AGP2, and Arg90 is substituted for a lysine which may lead to a loss of the optimal cation-π interaction UCN-01 as well as disrupting the orientation of the equivalent to Glu64 that H-bonds to C7-OH. In addition, the disulphide bridge that anchors the β−sheet bearing Glu64 appears to be absent in rat AGP1 which may also influence the precise orientation of this residue (32). In canine AGP1, binding to UCN-01 is reduced 60-fold, the full complement of cysteine residues is present, but Arg90 is again substituted for a lysine residue. The dynamic and structural interplay of AGP2/UCN-01 binding is clearly complex, especially in response to mutations. In this case, however, binding data are available for its related structures, staurosporine and UCN-02 (Fig. 5), which provide a robust structure-function series that does not require protein mutations or perturbations (28). The binding affinity of staurosporine that lacks the 7-hydroxy group shows an approximate 20-fold reduction in binding affinity (*K*_d_ = 88 nM ± 30 nM) corresponding to ∼6 to 8 kJ mol^−1^ difference in binding energy. The structure reveals that the hydroxyl group of UCN-01 is important and is oriented toward Glu64, forming a hydrogen bond and indirectly assisting with orienting Arg90. This key interaction would be lost with staurosporine, assuming a similar binding mode and consistent with loss of the H-bonding interaction and reduced-binding affinity. Reversal of the chirality at C7 to the *S*-configuration in UCN-02 has a more drastic effect, with >100-fold loss of affinity such that UCN-01 and 02 are essentially the eutomer and distomer pair, respectively. The 100-fold reduction with UCN-02 is of course too large for the loss of a hydrogen bond alone. However, the reversal of the stereochemistry at C7 not only breaks this hydrogen bonding, but is likely to invoke a clash with Phe49 (Fig. 5*B*) and a further shift from the optimal binding pose. This combination may account for the much greater loss in binding affinity for UCN-02.

### Comparison to UCN-01 bound to cell cycle proteins CDK2 and Chk1.

**Figure 6:**
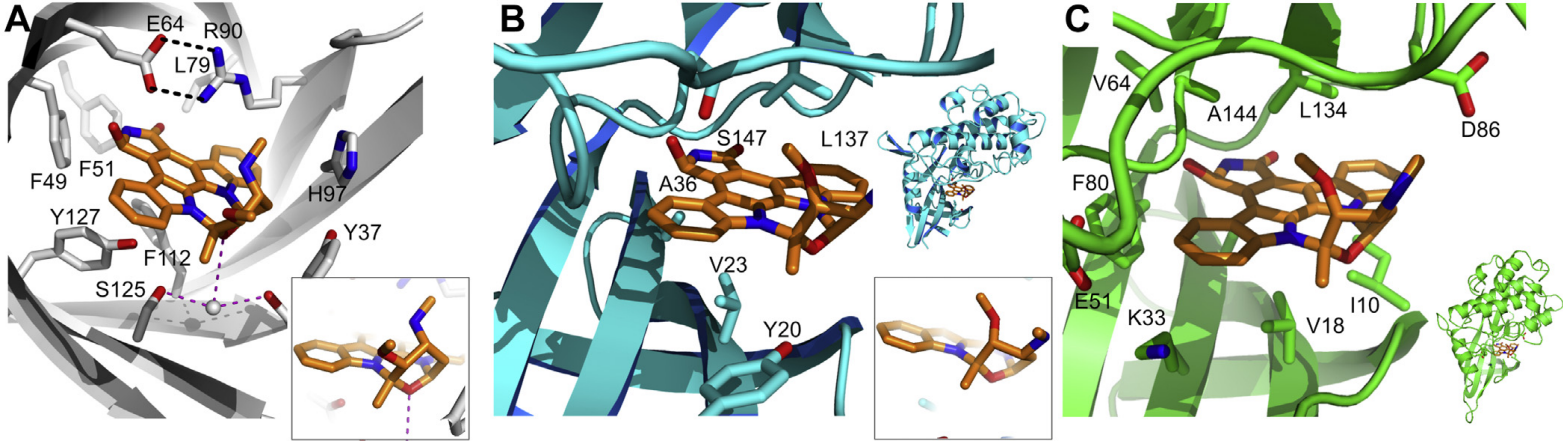
**Structural comparisons of high-resolution X-ray structures determined for UCN-01 bound to AGP2, Chk1, and CDK2.***A*, UCN-01 forms extensive hydrophobic contacts to AGP2 as it does with Chk1 and a hydrogen bond from the C7 hydroxyl group. The cation-π interaction to the aromatic system of UCN-01 is however distinct. The conformation of the pyran ring is highlighted as an inset (*B*) structure of the UCN-01-Chk1 binding pocket [PDB: 1NVQ]. The UCN-01 hydrophobic ring system is “sandwiched” in between hydrophobic residues of Chk1. The C7 hydroxyl group of UCN-01 forms a 2.86 Å hydrogen bond to Ser147. The alternate conformation of the pyran ring is highlighted as an inset (*C*) structure of CDK2 in complex with UCN-01 [PDB: 1PKD]. As with Chk1 hydrophobic residues on either side of the aromatic system, in this case, Val18 and Leu134 form binding contacts between ligand and protein. Unlike both AGP2 and Chk1, there are no residues near the hydroxy-lactam group to form H-bonds. AGP, alpha-1-acid glycoprotein; AGP2-FL, full-length AGP2; CDK2, cyclin dependent kinases; Chk1, Checkpoint 1.

UCN-01’s targets include the cell cycle proteins CDK2 and Chk1 for which high-resolution crystal structures have been determined (33, 34). Together with the AGP2–UCN-01 complex, these structures provide a structural framework for rational chemical modification and potentially abrogated AGP2 binding. Figure 6, *A* and *B* shows the structure of UCN-01 bound to AGP2 compared with the binding pocket of Chk1 aligned to UCN-01’s orientation. UCN-01 binding to Chk1 mimics adenine binding in terms of H-bonding and interaction with nonpolar surfaces. In fact, UCN-01 has greater potency as a Chk1 inhibitor than staurosporine (35) due to the presence of a hydrogen-bonding interaction between the C7 hydroxyl group in UCN-01 and Ser147 in much the same way that UCN-01 forms a favorable interaction with Glu64 in AGP2 (34). The aromatic system forming the central core of UCN-01 forms hydrophobic interactions with nonpolar residues in the binding pocket in both structures. In Chk1, these residues are Ala36, Val23, and Leu137. AGP2 uses a cation-π interaction between Arg90 and the ring on its basic face, but predominantly hydrophobic interactions on the reverse face as with the Chk1 complex. Interestingly, we observed a clear difference in conformation of the pyran ring of UCN-01 between the two complexes (Fig. 6 insets and Fig. S5). In the Chk1 complex, the pyran moiety is flipped with the methoxy and secondary amine moieties occupying axial and equatorial positions. This is reversed in the AGP2 complex, possibly to reduce clashes between the amine and Tyr37. UCN-01 also occupies a site between hydrophobic residues that surround both faces of UCN-01's aromatic system in the CDK2-binding cleft (Fig. 6*C*). In contrast to the binding mode of the compound in both AGP2 and Chk1, however, there are no hydrogen bond donors or acceptors proximal to the hydroxy-lactam l group of UCN-01. Again, the pyran ring conformation of UCN-01 differs in comparison with the AGP2 complex.

Therefore, UCN-01 is surrounded by a β-barrel in AGP2, whereas in Chk1 and CDK2, it is sandwiched between two β-sheets. It may therefore be possible to introduce substitutions on the UCN-01 aromatic system that protrude from these clefts and do not compromise binding Chk1/CDK2 binding affinity but preclude being enveloped in AGP2’s β-barrel. Alternatively, conformational restriction of the pyran ring could potentially prevent the methoxy or amine sidechains adopting the alternate conformation present, and perhaps, required for AGP2 binding.

## Conclusions

This study has presented the crystal structure of the important antitumour compound UCN-01 bound to AGP2, supplemented with backbone chemical shift assignments and relaxation parameters at 700 MHz. Our NMR studies provide the first indication that AGP2 is significantly more disordered in the free form than would have been assumed based on published crystal structures. The relaxation parameters, on the other hand, show that the main body of the protein displays mostly uniform dynamics when binding UCN-01 in solution. NMR spectral quality is also considerably improved in the presence of UCN-01 relative to the free form, consistent with conformational rigidification upon ligand binding.

The crystal structure reveals the binding mode of the highest known affinity ligand for AGP2 and rationalizes how the stereochemistry of the staurosporine hydroxy-lactam ring impacts binding affinity. These findings open new avenues for redesigning UCN-01 to maintain its antikinase activity, whereas avoiding high-affinity plasma binding which may generate useful leads toward new oncology therapeutics.

## Experimental procedures

### Expression, refolding, and purification of AGP2

AGP2-FL was initially expressed from a pOPINF (36) construct including residues 1 to 183 of the mature protein and a N-terminal hexa-his tag as follows: *MAHHHHHHSSG LEVLFQ*GPQIPLCANLVPVPITNATLDRITGKWFYIASAFR NEEYNKSVQEIQATFFYFTPNKTEDTIFLREYQTRQNQCF YNSSYLNVQRENGTVSRYEGGREHVAHLLFLRDTKTLMF GSYLDDEKNWGLSFYADKPETTKEQLGEFYEALDCLRIPR SDVMYTDWKKDKCEPLEKQHEKERKQEEGES. Eighteen N-terminal amino acid residues in italics were removed during purification by 3C proteolytic cleavage (see below). To assist in protein purification and expression, Cys149 of the native sequence was replaced with Arg as in AGP1 and published protocols (14). The truncated form of AGP2 was constructed by introducing a stop codon at Glu173 but was otherwise unchanged from the full-length pOPINF construct and purification and refolding protocols were identical. BL21 (DE3) cells were transformed with the construct and selected by ampicillin resistance. For nonisotopically labeled samples, cells were grown to absorbance ∼0.7 in LB growth media +2% glucose w/v at a 1:100 volumetric ratio. Alternatively for NMR studies, single (^15^N)- or double (^15^N and ^13^C)- labeled protein was produced from the cells grown to an absorbance ∼0.4 at 37 °C in isotope-labeled M9 minimal media containing 2.5 gl^−1^ ^13^C_6_-glucose and 1 gl^−1^ ^15^NH_4_Cl induced with 0.25 mM IPTG. The cells were harvested after 16 h at a reduced temperature of 30 °C. Inclusion bodies were prepared by lysing cells in hypoosmotic buffer (20 mM Tris, 50 mM NaCl, 1 mM EDTA, and 1 mM benzamidine, pH 8.0) and removing soluble material by centrifugation. The inclusion bodies were washed 2 times with lysis buffer and 2 to 3 times with high salt buffer (20 mM Tris, 500 mM NaCl, 1 mM EDTA, and 1 mM benzamidine, pH 8.0). The purified inclusion bodies were solubilized in denaturation buffer (50 mM tris and 8 M urea, pH 8.5) with a few drops of NaOH and stored (−20 °C) until further use.

For refolding, the solubilized inclusion bodies were reduced (10 mM DTT, 1 h, on ice), and excess insoluble material was removed by centrifugation (15 krpm, 10 min). The reduced denatured AGP2 was added dropwise to refolding solution (50 mM Tris, 1 M arginine, 3.5 mM cystamine, 6.5 mM cysteamine, 1 mM benzamidine, and 1 mM EDTA, pH 8.0) and incubated (48–72 h, 4 °C). The folded AGP2 was separated from impurities and denatured material by size-exclusion chromatography on S75 resin eluting at ∼215 ml postinjection (HiLoad 26/60, Amersham Biosciences). AGP2 was concentrated, and the His_6_-tag was removed by 3C proteolytic cleavage at 2 mg/ml with a 20:1 mass ratio of in-house 3C protease. The protease and His_6_-tag were removed by reverse IMAC to yield pure and cleaved AGP2.

Acetylation of AGP2 cysteine residues was performed by incubating a diluted ^15^N-labeled AGP2 NMR sample (50 μM and 50 μl) and iodoacetamide (15 mM) in the presence of absence of DTT (10 mM, 20 min, at 60 °C). The protein was recovered by methanol/chloroform precipitation.

### Crystallization and structure solution

0.5 mM AGP2 was incubated with 1 mM UCN-01 from a 50 mM DMSO stock (30 min, on ice), and the complex was crystallized in 100 mM HEPES and 1.4 M sodium acetate, pH7.4. A dataset of 1800 images was collected at a wavelength of 0.9686 Å, exposure time: 0.01 s, oscillation: 0.10° with cryogenic temperatures maintained throughout data acquisition. The dataset was scaled and merged into P61 using the Diamond Pipeline (Xi2 3dii) with cell dimensions of a = 88.27, b = 88.27, and c = 53.84 Å (α = 90 β = 90 γ = 120°). There was a single molecule in the asymmetric unit with a solvent content of 60%. The structure of AGP2 was determined by molecular replacement with MOLREP (37) within the CCP4i2 suite (38) using the human α_1_-acid glycoprotein model (3APU). The resulting electron density maps were of sufficient quality to autobuild 90% of the model. Iterative rounds of manual rebuilding and refinement in Coot (39) and Phenix.refine (40) further improved the Rfree values before the final structure was validated with MolProbility (41). The final statistics are provided in Table 1.

**Table 1:** Data collection and refinement statistics for AGP2 in complex with UCN-01

Wavelength (Å)	0.9686 Å
Resolution range (Å)	44.13–1.82 (1.885–1.82)
Space group	P 61
*a*, *b*, *c* (Å)	88.27 88.27 53.84
*α*, *β*, γ (°)	90.0 90.0 120.0
Total reflections	395,322 (23,753)
Unique reflections	21,515 (2112)
Multiplicity	18.4 (11.2)
Completeness (%)	99.72 (97.69)
Mean I/sigma (I)	34.31 (11.22)
Wilson B-factor (Å^2^)	21.45
R-merge	0.05843 (0.149)
R-meas	0.06 (0.1561)
R-pim	0.01354 (0.0455)
CC1/2	0.999 (0.991)
CC∗	1 (0.998)
Reflections used in refinement	21,514 (2112)
Reflections used for R-free	986 (118)
R-work	0.1582 (0.1926)
R-free	0.1765 (0.2221)
CC (work)	0.972 (0.911)
CC (free)	0.961 (0.917)
Number of nonhydrogen atoms	1722
Macromolecules	1448
Ligands	36
Solvent	238
Protein residues	173
R.M.S. deviations	
Bond lengths (Å)	0.007
Bond angles (°)	1.17
Ramachandran favored (%)	99.42
Ramachandran allowed (%)	0.58
Ramachandran outliers (%)	0.00
Rotamer outliers (%)	0.00
Clashscore	2.42
B factors (Å^2^)	
Average	30.48
Macromolecules	29.21
Ligands	19.13
Solvent	39.96
Number of TLS groups	8

Statistics for the highest-resolution shell are shown in parentheses.

### CD spectroscopy

AGP2 was exchanged into 20 mM NaP and 150 mM NaF, pH 7.2 by Zeba column. The CD experiments were acquired on an Applied Photophysics Chirascan instrument with the manufacturer software v. 4.7.0.194.

### NMR chemical shift assignment experiments

To assess protein quality, ^1^H-^15^N TROSY-HSQC spectra were collected on a 700 MHz Bruker AVANCE^*HD*^ NMR spectrometer equipped with a 1.7 mm triple-resonance micro-cryoprobe. The samples were composed of 10 mM Na_2_HPO_4_/NaH_2_PO_4_ and 100 mM NaCl, pH 6.5 with 0.5 or 1 mM AGP2 with at least a 2-fold excess UCN-01 when required. Although the AGP2 samples were not perdeuterated, TROSY-HSQC spectra were collected for assessment of protein folding to allow higher ^1^H dimension resolution (>4K complex points) without delivering decoupling power to the probe. The chemical shift assignments for 98% of the nonproline backbone residues were obtained by manual analysis of ^1^H-^15^N TROSY-HSQC, HNCA, HNCOCA, HNCACB, and HNCOCACB spectra collected at 700 MHz and ^1^H-^15^N HSQC-NOESY, ^1^H-^13^C HSQC-NOESY, and CBCANH acquired at 950 MHz using standard Bruker pulse sequences.

### NMR Titrations of AGP2 with UCN-01.

In a reverse minimal shift approach, peaks in the free spectrum were assigned based on their proximity to assigned peaks in the UCN-01-bound spectrum. The free peaks closest to liganded peaks were assigned first, and subsequently, more distant free peaks were assigned to the closest remaining unassigned UCN-01 peak provided that the peaks were closer than 0.1 ppm in the ^1^H dimension and 0.68 ppm in the ^15^N dimension. Heteronuclear shifts were weighted based on the respective ranges of assigned peak chemical shifts present in the two dimensions (^1^H:4.504 and ^15^N:30.616), as described in published methods (42). These ranges yielded a weighting of 0.147 ^1^H ppm/^15^N ppm. All the visible assigned peaks in the UCN-01 spectrum were greater than 5% of the height of the largest peak (His172) and so, signals in the free spectrum were only counted if they were more than 5% of the height of the largest free AGP2 resonance (putative His172).

#### Relaxation measurements

Longitudinal relaxation times (*T*_1_) were measured using the standard Bruker sequence, tr1etf3gpsi, with relaxation delays of 10, 50, 100, 200, 400, 600, 800, 1000, 1200, 1500, 1800, 2000, and 2500 ms and a recovery delay of 3 s. Transverse relaxation times (*T*_2_) were measured using the standard Bruker sequence, tr2etf3gpsi, with relaxation delays of 10, 48, 64, 80, 96, 128, 160, 192, 240, and 320 ms and a recovery delay of 1.5 s. For ^15^N steady-state NOE experiments, the data were acquired interleaved using the standard Bruker sequence, trnoef3gpsi, with a saturation period of 6 s. The spectra were processed with NMRPipe (43) and analyzed with CcpNMR Analysis Version 2.4.2 (44).

## Data availability

The final model of AGP-2 bound to UCN-01 has been deposited to the PDB with accession code 7OUB. The chemical shift assignments have been deposited in the BMRB with accession number 50946.

## Supporting information

This article contains supporting information (45).

## Conflict of interest

The authors declare that they have no conflicts of interest with the contents of this article.
