# Supplementary material for: The structural basis for high affinity binding of α1-acid glycoprotein to the potent antitumor compound UCN-01
Source: J Biol Chem. 2021 Nov 7;297(6):101392. doi: 10.1016/j.jbc.2021.101392 (PMC8671939; doi:10.1016/j.jbc.2021.101392)
Supplement: Figures S1–S5 [file mmc1.pdf]

Electronic Supporting Information

The structural basis for high affinity binding of α1-acid Glycoprotein to the potent anti-tumour compound UCN-01

Erik J. B. Landin[1], Christopher Williams[1,2], Sara A. Ryan[1], Alice Bochel[1], Nahida Akter[1], Christina Redfield[3], Richard B. Sessions[4], Neesha Dedi[5], Richard J. Taylor[5], Matthew P. Crump[1,*]

[1]School of Chemistry, Cantock's Close, University of Bristol, Bristol BS8 1TS, UK

[2]BrisSynBio, Life Sciences Building, Tyndall Avenue, Bristol, BS8 1TQ, UK

[3]Department of Biochemistry, University of Oxford, South Parks Road, Oxford, OX1 3QU, UK

[4]School of Biochemistry University of Bristol, University Walk, BS8 1TD, Bristol, UK

[5]Discovery Sciences, UCB Biopharma, 216 Bath Road, Slough, SL1 3WE, UK

*To whom correspondence should be addressed: E-mail: matt.crump@bristol.ac.uk; Richard.taylor@ucb.com

Figure S1. Comparison of ^{1}H-^{15}N TROSY-HSQC spectra of comparison of free and UCN-01 bound AGP2-FL. (A) ^{1}H-^{15}N TROSY-HSQC solution spectrum of AGP2-FL (0.5 mM) at pH 6.5 in 10 mM Na_2HPO_4/NaH_2PO_4, 100 mM NaCl buffer. Significant peak dispersion was consistent with the presence of folded protein. 119 significant resonances were observed; this count is substantially lower than the expected 169 resonances expected for this construct (166 backbone peaks and 3 Trp indole ring NH signals) and so it was concluded that exchange broadening was reducing the intensity of a significant portion of the signals. (B) ^{1}H-^{15}N TROSY-HSQC of AGP2-FL (0.5 mM) in the presence of 2-fold excess of UCN-01. 165 significant resonances were present out of a total 179 expected resonances. Spectra were collected at 700 MHz. (C) Analytical S75 trace for AGP2-FL with an elution time of 12 ml and estimated M_w of 22 kDa (expected 21854 Da for monomer). Calibration curve calculated for analytical S75 column where $K_{av}= V_e\text{-}V_o/V_t\text{-}V_o$ where V_e is the elution volume, V_o the void volume (7.14 ml) and V_t the column volume (24.00 ml). Calibrants were conalbumin (Mw 75 kDa, Ve 9.02 ml), ovalbumin (44 kDa, 9.78 ml), carbonic anhydrase (29 kDa, 11.13 ml), ribonuclease A (13.70 kDa, 13.07 ml).

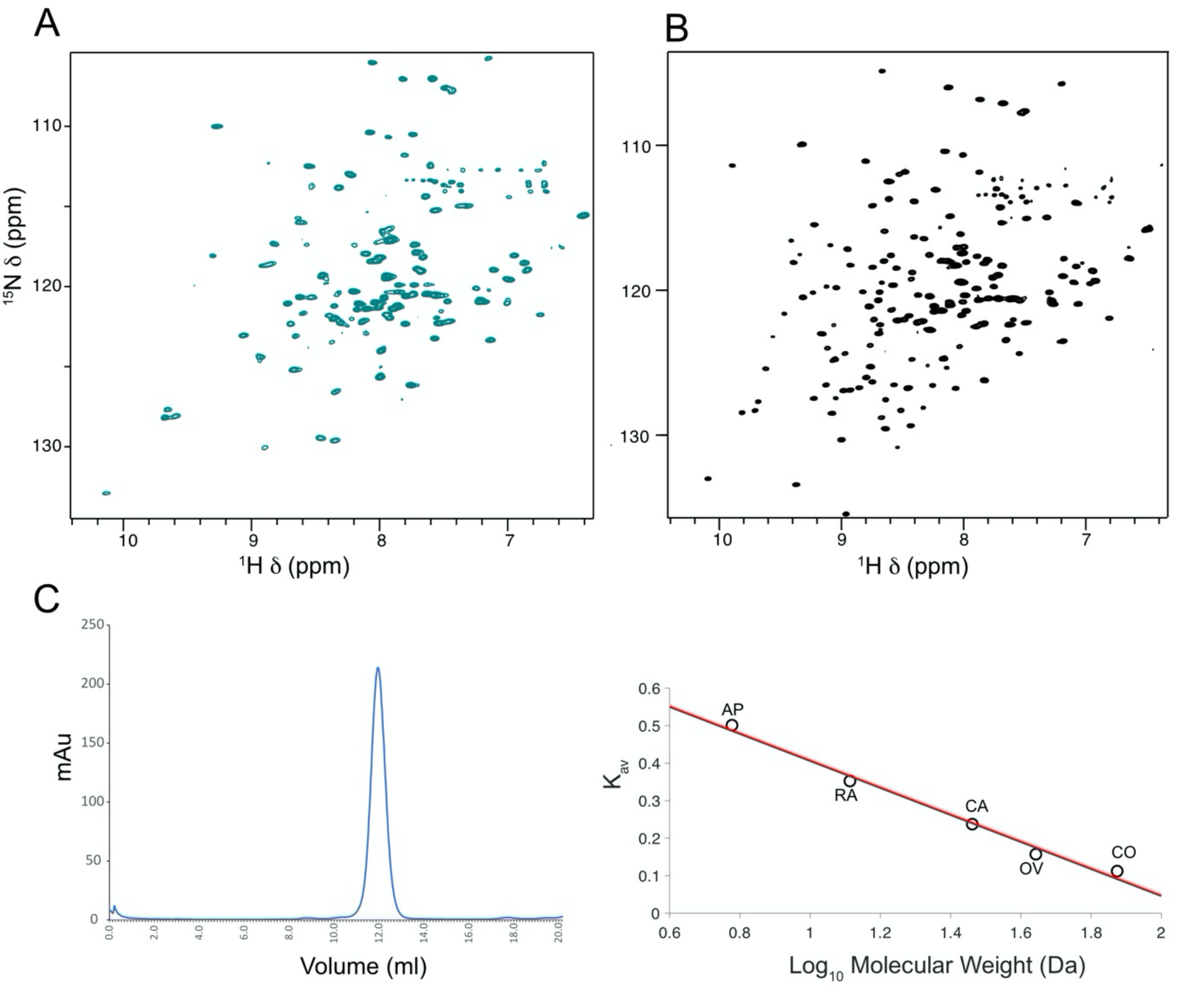

Figure S2 Purification and characterisation of AGP2.

(A) AGP2 sequence used in these structural studies. This includes the C-terminal truncation (so expressed AGP2 sequence terminates at His172) and an N-terminal His_6-tag with a 3C protease cleavage site for post-expression removal of the His_6-tag.

(B) SDS PAGE gel of AGP2 N-terminal 3C protease cleavage reaction. The protein was cleaved and yielded a band (lane three) consistent with pure cleaved protein after negative purification; S75 analytical size exclusion chromatography trace indicated monomeric protein.

(C) ESMS trace of purified AGP2 (observed 20528.6 Da, expected 20524 Da).

(D) Deconvoluted (top) and raw (bottom) ESI-MS spectra of purified ^{15}N-labelled AGP2. Left - untreated AGP2 yielded a peak at 20762 compared to the expected mass of 20769 assuming 100% ^{15}N labelling. Middle - treated with IAA without DTT resulted in no change indicating that all four thiol groups were oxidised and in disulphide bonds. Right - spectrum of AGP2 reduced with IAA and DTT acted as a positive control with a mass increase of 231 to a total of 20922 Da corresponding to the addition of four 58 Da acetamide groups. The raw ESI-MS spectrum also showed a shift to higher m/z values indicative of greater exposure of ionisable side chains and unfolding after disulphide bonds are reduced.

(E) CD melt traces of AGP2 without ligand (left, T_M 61.9 ± 0.1 °C) and in the presence of a 2-fold excess of UCN-01 (right, T_M 75.6 ± 0.2 °C).

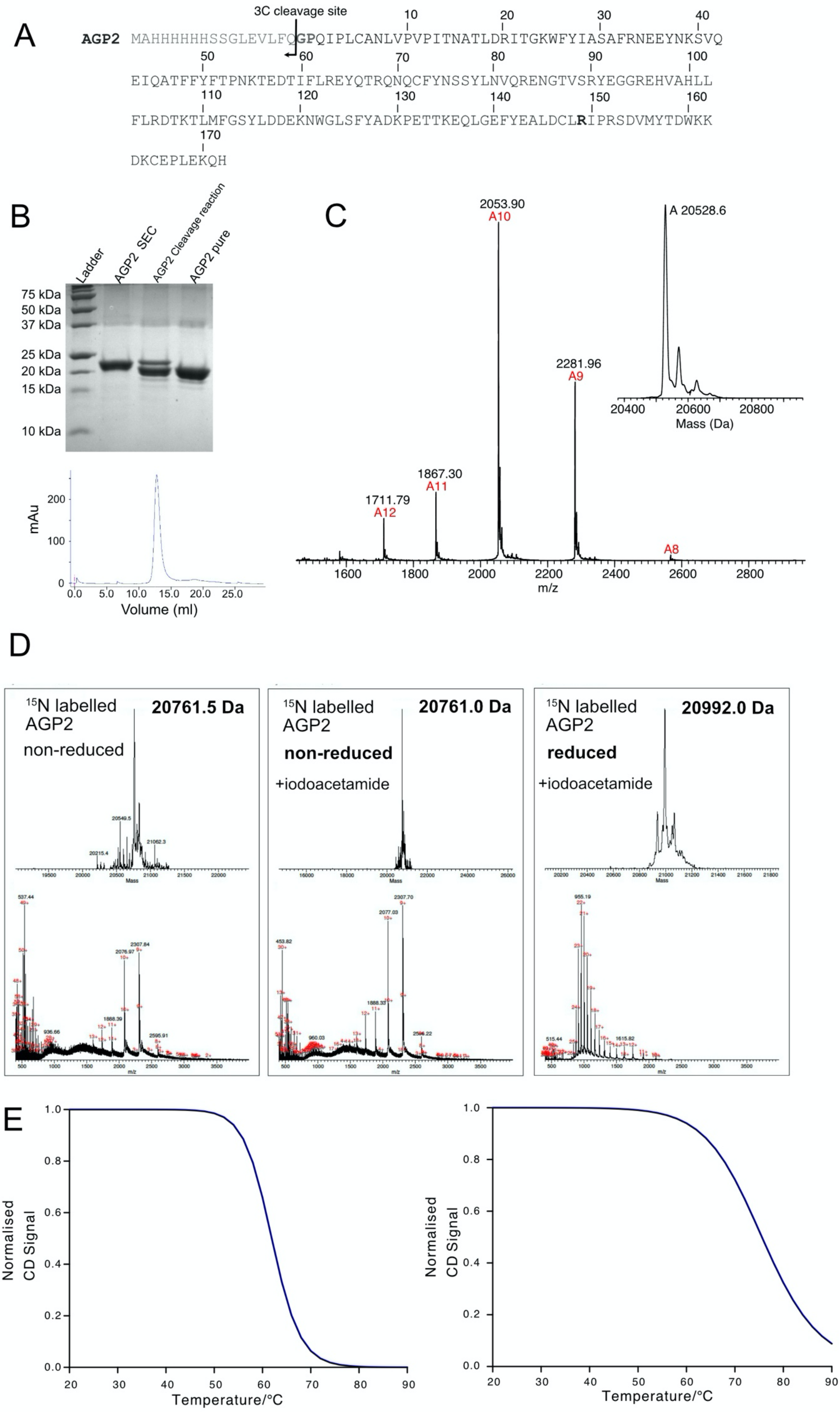
A
AGP2
3C cleavage site
MAHHHHHHSSGLEVLFQGPQIPLCANLVPVPITNATLDRITGKWFYIASAFRNEEYNKSVQ
EIQATFFYFTPNKTEDTIFLREYQTRQNQCFYNSSYLNVQRENGTVSRYEGGREHVAHLL
FLRDTKTLMFGSYLDDEKNWGLSFYADKPETTKEQLGEFYEALDCLRIPRSDVMYTDWKK
DKCEPLEKQH
B
Ladder
AGP2 SEC
AGP2 Cleavage reaction
AGP2 pure
75 kDa
50 kDa
37 kDa
25 kDa
20 kDa
15 kDa
10 kDa
mAu
Volume (ml)
C
2053.90
A10
2281.96
A9
1867.30
A11
1711.79
A12
A8
m/z
A 20528.6
Mass (Da)
D
^{15}N labelled AGP2
non-reduced
20761.5 Da
^{15}N labelled AGP2
non-reduced
+iodoacetamide
20761.0 Da
^{15}N labelled AGP2
reduced
+iodoacetamide
20992.0 Da
E
Normalised CD Signal
Temperature/°C
Normalised CD Signal
Temperature/°C

Figure S3 NMR monitoring of changes between apo and UCN-01 bound AGP2. (A) Overlay of apo (red) and 1:2 protein:UCN-01 (blue) spectra at 700 MHz indicates significant conformational changes occur upon ligand binding. Signals for residues involved in binding such as Arg90 and Phe112, which are absent in the apo spectrum, are revealed on ligand binding, indicating that the binding site is rigidified to accommodate ligand. Further, other residues not directly involved in binding, such as Leu101 are also not resolved in the apo spectrum, indicating that a structural re-arrangement and rigidification remote from the binding site also occurs. (B) Structure of AGP2 with residues coloured based on whether backbone N-H peaks are observed (red) or not (blue) in the apo spectrum based on the UCN-01 spectrum. Proline residues are highlighted black and residues only visible at different field strengths are highlighted in pink. (C) Quantitative map of chemical shift perturbations, between apo and UCN-01 bound NMR spectra. A value of 0 indicates a peak not located in the apo spectrum.

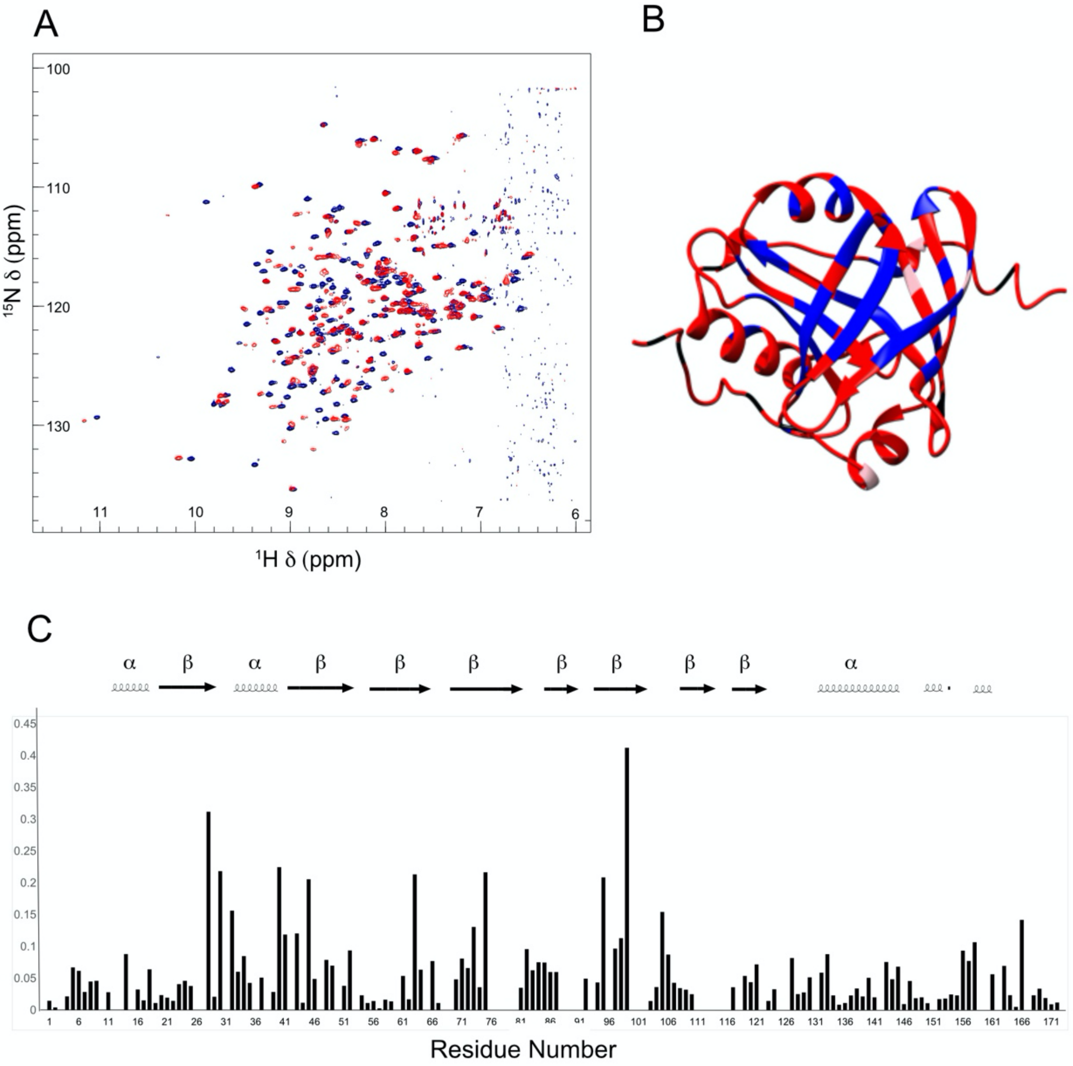

Figure S4 Relaxation and NOE parameters for backbone residues of AGP2 bound to UCN-01 at 700 MHz. A) ^{15}N-T_1, B) ^{15}N-T_2 and C) NOE recorded at 700 MHz. Elements of secondary structure are shown as determined by STRIDE (Frishman and Argos 1995). Proline residues are marked with black bars.

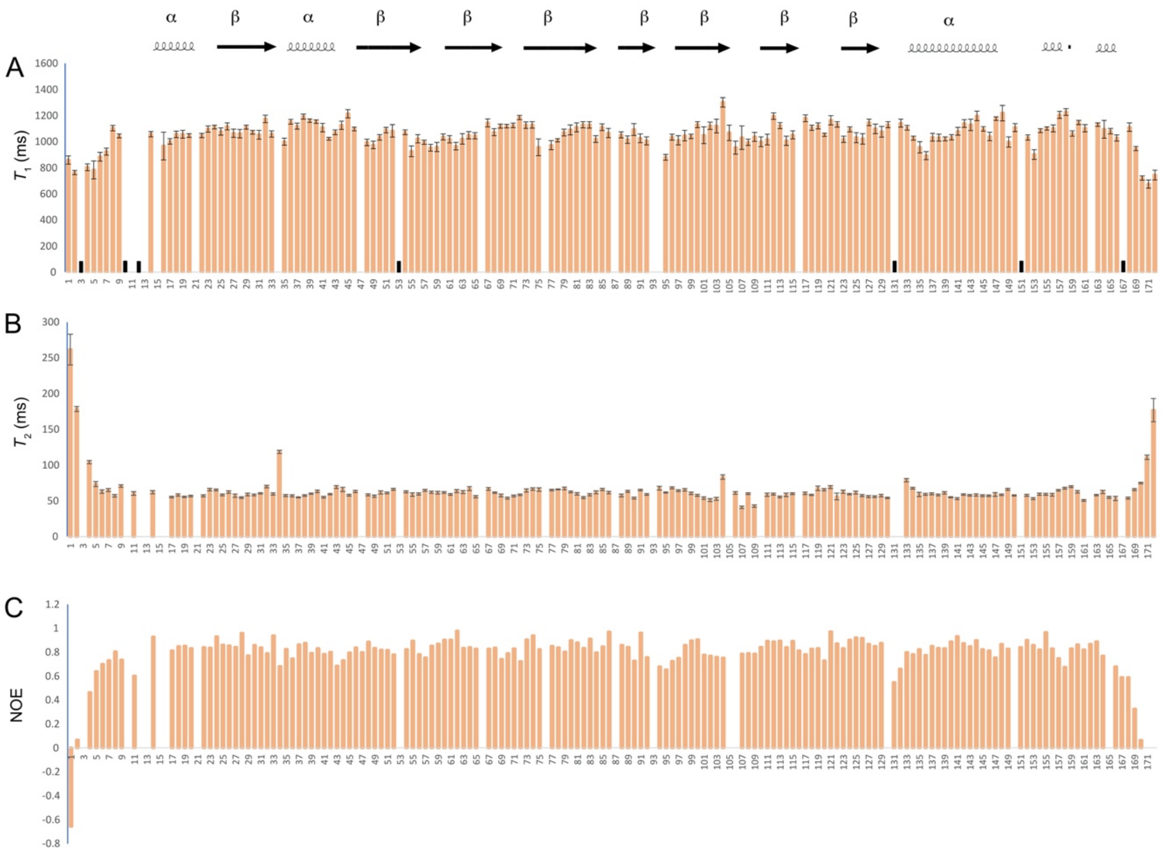

Figure S5. **Electron density of UCN-01 bound to AGP2.** (A) The unbiased composite OMIT map contoured at 2.5σ generated in Phenix version 1.19.2 shows well-defined density (>3σ) in the binding pocket covering all the UCN-01 atoms. (B) Enlarged view of the UCN-01 binding pocket of AGP2. UCN-01 could not be modelled into the binding site using the representative database model, instead the ideal conformation was used. Close inspection of the binding pocket shows that the methoxy and secondary amine groups adopt the ideal conformation to prevent clashing with Tyr37 of AGP2. The density map is contoured at 2.5σ.

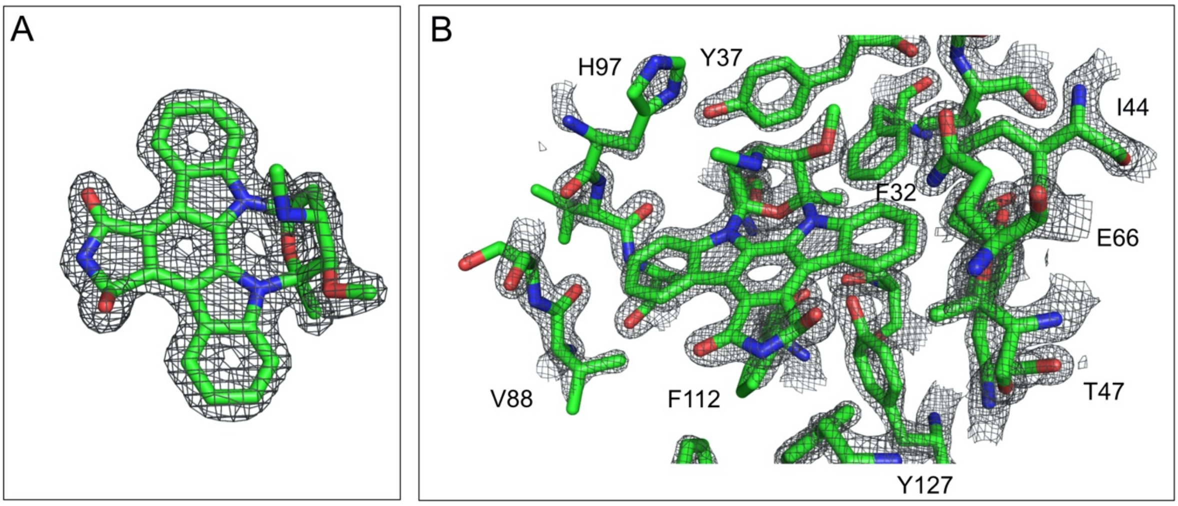

References

Frishman, D., and P. Argos. 1995. 'Knowledge-based protein secondary structure assignment', *Proteins Struct. Funct. Genet.*, 23: 566-79.
